# Characterization of *Staphylococcus aureus* Primosomal DnaD Protein: Highly Conserved C-Terminal Region Is Crucial for ssDNA and PriA Helicase Binding but Not for DnaA Protein-Binding and Self-Tetramerization

**DOI:** 10.1371/journal.pone.0157593

**Published:** 2016-06-15

**Authors:** Yen-Hua Huang, Yi Lien, Chien-Chih Huang, Cheng-Yang Huang

**Affiliations:** 1 School of Biomedical Sciences, Chung Shan Medical University, No.110, Sec.1, Chien-Kuo N. Rd., Taichung City, Taiwan; 2 Department of Medical Research, Chung Shan Medical University Hospital, No.110, Sec.1, Chien-Kuo N. Rd., Taichung City, Taiwan; Saint Louis University, UNITED STATES

## Abstract

The role of DnaD in the recruitment of replicative helicase has been identified. However, knowledge of the DNA, PriA, and DnaA binding mechanism of this protein for the DnaA- and PriA-directed replication primosome assemblies is limited. We characterized the DNA-binding properties of DnaD from *Staphylococcus aureus* (SaDnaD) and analyzed its interactions with SaPriA and SaDnaA. The gel filtration chromatography analysis of purified SaDnaD and its deletion mutant proteins (SaDnaD1-195, SaDnaD1-200 and SaDnaD1-204) showed a stable tetramer in solution. This finding indicates that the C-terminal region aa 196–228 is not crucial for SaDnaD oligomerization. SaDnaD forms distinct complexes with ssDNA of different lengths. In fluorescence titrations, SaDnaD bound to ssDNA with a binding-site size of approximately 32 nt. A stable complex of SaDnaD1-195, SaDnaD1-200, and SaDnaD1-204 with ssDNA dT40 was undetectable, indicating that the C-terminal region of SaDnaD (particularly aa 205–228) is crucial for ssDNA binding. The SPR results revealed that SaDnaD1-195 can interact with SaDnaA but not with SaPriA, which may indicate that DnaD has different binding sites for PriA and DnaA. Both SaDnaD and SaDnaDY176A mutant proteins, but not SaDnaD1-195, can significantly stimulate the ATPase activity of SaPriA. Hence, the stimulation effect mainly resulted from direct contact within the protein—protein interaction, not via the DNA—protein interaction. Kinetic studies revealed that the SaDnaD-SaPriA interaction increases the *V*_max_ of the SaPriA ATPase fivefold without significantly affecting the *K*_m_. These results indicate that the conserved C-terminal region is crucial for ssDNA and PriA helicase binding, but not for DnaA protein-binding and self-tetramerization.

## Introduction

Initiation and re-initiation of chromosomal DNA replication in bacteria is a complex process that depends on divergent multi-protein assembly for loading the replicative DNA helicase at the replication origin [1,2,3,4,5]. DNA damage causes arrest and disassembly of the replication machinery, anywhere along the DNA, leading to replication failure [6,7,8]. Genome integrity should be maintained from generation to generation to ensure proper cell function and survival; thus, collapsed DNA replication forks must be reactivated through origin-independent replisome reloading for genome duplication. The replication restart primosome [9,10,11] is a formidable enzymatic machine; it is a protein—DNA complex that reactivates stalled DNA replication at repaired replication forks after DNA damage [12]. In contrast to the DnaA-directed primosome initiated at the unique *oriC* site [3,4], the replication restart primosome preferentially recognizes three-way branched DNA structures that possess a leading strand [13,14,15,16].

The replication restart primosome in *Escherichia coli* includes seven essential proteins, namely, PriA helicase, PriB, PriC, DnaB helicase, DnaC, DnaT, and DnaG primase [10]. The mechanisms of action of DNA replication restart primosome in bacteria have mainly been studied in Gram-negative *E*. *coli*, and, to a lesser extent, in Gram-positive bacteria [17]. Different bacteria have different strategies for the functional loading of cellular replicative DNA helicases [18]. In the Gram-positive *Bacillus subtilis*, the DNA replication initiator protein PriA helicase has a homolog of *E*. *coli* [19]. Nevertheless, essential helicase-loading components, such as PriB, PriC, DnaT, and DnaC proteins, are not found in Gram-positive bacteria [20]. Instead, three other proteins, namely, DnaD, DnaB, and DnaI, have been genetically and biochemically proven to be required for the replication restart of the Gram-positive *B*. *subtilis* [21,22,23,25,26]. The DnaD and DnaB have no homologs in Gram-negative bacteria, and their functions for DnaA- and PriA-dependent initiation of DNA replication need to be examined.

DnaD interacts with DnaA [26], DnaB [24], and DnaD itself [27]. Following DnaA assembly at *oriC* in *B*. *subtilis*, DnaD and DnaB are sequentially recruited and are required to deposit the protein complex of the helicase loader DnaI and the DNA helicase DnaC onto the unwound DNA duplex [28]. DnaD inhibits the cooperative DNA-binding activity [29], and may regulate DNA replication initiation. DnaD forms scaffolds and enhances duplex melting [30]. DnaD functions as a global regulator of DNA architecture [22] and a potential modulator for global superhelical density [31]. Moreover, this protein interacts with linear DNA and forms a nucleoprotein structure with a round DnaD scaffold in an open circle [32]. DnaD consists of an N-terminal domain with oligomerization activity and a C-terminal domain with single-stranded DNA (ssDNA)-binding activity [27]. The crystal structure of the N-terminal domain of DnaD reveals an extended winged-helix fold [33] and a unique tetramerization motif for the DnaD-mediated scaffold formation [34]. NMR analysis of the structure of the DnaD C-terminal domain reveals the presence of five helices [35]. Although DnaD can bind to DNA and form large nucleoprotein complexes in the absence of the N-terminal domain, it does not exhibit DNA remodeling activity [27].

DnaD is a component of the DNA replication primosome, but information on PriA and DnaA binding of DnaD is limited. Knowledge of stoichiometry, domain function, and binding activity is a prerequisite for formulating any model of protein function in DNA replication. In this study, the DNA-DnaD interactions were analyzed through electrophoretic mobility shift analysis (EMSA) and fluorescence quenching. Furthermore, considering our collective data from surface plasmon resonance (SPR) experiments, ATPase stimulation effects and gold nanoparticle assays on DnaD—PriA and DnaD—DnaA interactions, as well as studies on deletion mutants, we propose and discuss probable domain functions of DnaD.

## Materials and Methods

### Construction of SaDnaD, SaDnaD1-195, SaDnaD1-200, SaDnaD1-204, SaPriA, and SaDnaA expression plasmids

The gene encoding the putative *Staphylococcus aureus* PriA (SaPriA), SaDnaA, and SaDnaD was individually amplified by PCR using the genomic DNA of *S*. *aureus* subsp. *aureus* ED98 as template. The forward and reverse primers were designed to introduce unique restriction sites into SaDnaD and its deletion mutants, permitting the insertion of the amplified gene into the pET21e vector for protein expression in *E*. *coli*. SaDnaA gene was amplified and inserted into the pET21b vector. Construction of the SaPriA expression plasmid has been reported [36]. The pET21e vector [37] was engineered from the pET21b vector (Novagen Inc., Madison, WI, USA) to avoid N-terminal T7 tag. Primers used for the construction of these plasmids are listed in Table 1.

**Table 1:** Primers used for construction of plasmids.

Oligonucleotide	Primer
SaDnaD-EcoRI-N	GGGGAATTCGATAAATATCAATTAAAA
SaDnaD-HindIII-C	GGGAAGCTTCTTACCATCAAGGTTCTC
SaDnaD1-204-EcoRI-N	GGGGAATTCGATAAATATCAATTAAAA
SaDnaD1-204-HindIII-C	GGGAAGCTTTTTATTAAATTTTTCTCT
SaDnaD1-200-EcoRI-N	GGGGAATTCGATAAATATCAATTAAAA
SaDnaD1-200-HindIII-C	GGGAAGCTTTTCTCTTATTTTTCTAGA
SaDnaD1-195-EcoRI-N	GGGGAATTCGATAAATATCAATTAAAA
SaDnaD1-195-HindIII-C	GGGAAGCTTAGAATCGTCAATTGTTTT
SaDnaA-NdeI-N	GGGCATATGTCGGAAAAAGAAATTTGG
SaDnaA-XhoI-C	GGGCTCGAGTACATTTCTTATTTCTTT

These plasmids were verified by DNA sequencing. Underlined nucleotides indicate the designated site for the restriction site.

### Protein expression and purification

The recombinant proteins were expressed and purified using the protocol described previously for PriB [38]. Briefly, *E*. *coli* BL21(DE3) cells were transformed with the expression vector and overexpression of the expression plasmids was induced by incubating with 1 mM isopropyl thiogalactopyranoside. The protein was purified from the soluble supernatant by Ni^2+^-affinity chromatography (HiTrap HP; GE Healthcare Bio-Sciences), eluted with Buffer A (20 mM Tris-HCl, 250 mM imidazole, and 0.5 M NaCl, pH 7.9), and dialyzed against a dialysis buffer (20 mM HEPES and 100 mM NaCl, pH 7.0; Buffer B). Protein purity remained at >97% as determined by SDS-PAGE (Mini-PROTEAN Tetra System; Bio-Rad, CA, USA).

### Gel-filtration chromatography

Gel-filtration chromatography was carried out by the AKTA-FPLC system (GE Healthcare Bio-Sciences, Piscataway, NJ, USA). In brief, purified protein (2 mg/mL) in Buffer B was applied to a Superdex 200 HR 10/30 column (GE Healthcare Bio-Sciences, Piscataway, NJ, USA) equilibrated with the same buffer. The column was operated at a flow rate of 0.5 mL/min, and the proteins were detected at 280 nm. The column was calibrated with proteins of known molecular weight: thyroglobulin (670 kDa), γ-globulin (158 kDa), ovalbumin (44 kDa), and myoglobin (17 kDa). The *K*_av_ values for the standard proteins and the SaDnaD variants were calculated from the equation: *K*_av_ = (*V*_e_ − *V*_o_)/(*V*_c_ − *V*_o_), where *V*_o_ is the column void volume, *V*_e_ is the elution volume, and *V*_c_ is the geometric column volume.

### EMSA

EMSA for the SaDnaD variants was conducted using the protocol described previously for PriB [39]. In brief, radiolabeling of various lengths of ssDNA oligonucleotides was carried out with [γ^32^P]ATP (6000 Ci/mmol; PerkinElmer Life Sciences, Waltham, MA) and T4 polynucleotide kinase (Promega, Madison, WI, USA). Protein (0, 0.06, 0.12, 0.25, 0.5, 1, 2, 4, 8, and 16 μM; monomer) was incubated at 25°C for 30 min with 1.7 nM DNA substrates (dT20–65) in a total volume of 10 μL in 20 mM Tris—HCl (pH 8.0) and 100 mM NaCl. The resulting samples were mixed with gel-loading solution (0.25% bromophenol blue and 40% sucrose; w/v), resolved on a native 8% polyacrylamide gel (8.3 × 7.3 cm) at 4°C in TBE buffer for 1–1.5 h at 100 V, and were visualized by phosphorimaging. The phosphor storage plate was scanned, and the data for complex and free DNA bands were digitized for quantitative analysis. The ssDNA-binding ability for the protein was estimated using linear interpolation from the protein concentration that binds 50% of the input DNA [40]. Each [Protein]_50_ was calculated as the average of at least three measurements ± S.D.

### Preparation of dsDNA substrates

The double-stranded DNA substrates (dsDNA) were prepared with a radiolabeled PS4 strand (3′-GGGCTTAAGCCTATCGAGCCATGGG-5′; 25 mer) and an unlabeled PS3-dT25 strand (5′-CCCGAATTCGGATAGCTCGGTACCC-dT25-3′) at a 1:1 concentration ratio. Each dsDNA substrate was formed in 20 mM HEPES (pH 7.0) and 100 mM NaCl, by brief heating at 95°C for 5 min and then followed by slow cooling to room temperature overnight.

### Fluorescence-quenching measurement

Fluorescence titrations were performed in a spectrofluorimeter (Hitachi F-2700; Hitachi High-Technologies, Tokyo, Japan) as described previously [41]. The excitation and emission of tryptophan fluorescence were detected at 295 and 340 nm, respectively. The protein solution (0.1 μM; tetramer) in 2 mL Tris—HCl buffer (20 mM Tris—HCl, and pH 8.0) containing 200 or 500 mM NaCl was titrated with increasing quantities of dT65 oligonucleotide. After the addition of ssDNA, the complex solution was equilibrated for 300 s until no fluorescence change could be observed. Tryptophan fluorescence quenching was used to detect and quantify protein-DNA interactions.

### SPR

SPR was conducted using the protocol described previously for DnaC helicase [42]. SaPriA and SaDnaA were individually immobilized on Series S sensor chips CM5 (GE Healthcare Bio-Sciences, Piscataway, NJ, USA). The PriA and DnaA-binding experiments were carried out at 293K using a Biacore T200 (GE Healthcare Bio-Sciences, Piscataway, NJ, USA) with running buffer (40 mM Tris, 200 mM NaCl, and 0.05% Tween-20 at pH 8.0). SaDnaD solutions were diluted in the running buffer to final concentrations of 250, 125, 63, 31, 15, and 7.6 nM. Diluted samples were injected in duplicate over each immobilized protein for 120 s at a flow rate of 30 μL/min. The running buffer was then flushed for 300 s at a flow rate of 30 μL/min. Finally, the chip surface was regenerated by injecting 2 M MgCl_2_ buffer for 60 s at a flow rate of 30 μL/min. Control samples were used to monitor the sensor chip surface stability, demonstrating reproducibility throughout the duration of the experiments. The estimated *K*_d_ values were derived by fitting the association and dissociation signals with a 1:1 (Langmuir) model using the Biacore T200 Evaluation Software.

### Gold nanoparticle assay

The protein—protein interactions within SaDnaD—SaPriA and SaDnaD—SaDnaA were rapidly analyzed based on the intrinsic optical properties of the gold nanoparticles [43]. The commercialized kit of Ni^2+^-NTA SAM nanoparticles (SmarticleParticles) was purchased from Minerva Biotechnologies (Waltham, MA, USA). The 75-μL coupling sample 1 contains 50 μL of SaDnaD (1 μM) and 25 μL of Ni^2+^-NTA SAM nanoparticles, whereas the 75-μL coupling sample 2 contains 50 μL of SaPriA (1 μM) or SaDnaA (1 μM) and 25 μL of Ni^2+^-NTA SAM nanoparticles. Sample 1 was mixed with sample 2 for 1–10 min. Red/pink indicates a homogeneous suspension, and purple indicates that particles are drawn close together through protein—protein interactions.

### ATPase assay

SaPriA ATPase assay was performed with 0.4 mM [γ-32P] ATP and 0.125 μM of SaPriA in reaction buffer containing 40 mM Tris (pH 8.0), 10 mM NaCl, 2 mM DTT, 2.5 mM MgCl_2_, and 0.1 μM PS4/PS3-dT25 DNA substrate. Aliquots (5 μL) were taken and spotted onto a polyethyleneimine cellulose thin-layer chromatography plate, which was subsequently developed in 0.5 M formic acid and 0.25 M LiCl for 30 m. Reaction products were visualized by autoradiography and quantified with a phosphorimager. The kinetic parameters *K*_m_ and *V*_max_ were determined from a non-linear plot by fitting the hydrolyzing rate from individual experiments to the Michaelis—Menten equation (Enzyme Kinetics module of Sigma-Plot; Systat Software, Chicago, IL, USA) [44].

### Cloning, protein expression, and purification of tag-free SaDnaD, SaDnaDY176A, and SaDnaD1-195 proteins

To obtain proteins without a His tag, SaDnaD and SaDnaD1-195 were amplified through PCR and cloned in a pGEX-5X-1 vector (GE Healthcare Bio-Sciences, Piscataway, NJ, USA) by using the restriction sites EcoRI and XhoI. SaDnaDY176A mutant was generated using a QuikChange Site-Directed Mutagenesis kit according to the manufacturer’s protocol (Stratagene, LaJolla, CA). These expression plasmids were verified by DNA sequencing. Table 2 lists the primers utilized to construct these GST plasmids.

**Table 2:** Primers used for construction of GST plasmids.

Oligonucleotide	Primer
GST-SaDnaD-EcoRI-N	GGGGAATTCGATAAATATCAATTAAAA
GST-SaDnaD-XhoI-C	GGGCTCGAGTTACTTACCATCAAGGTT
GST-SaDnaD1-195-EcoRI-N	GGGGAATTCGATAAATATCAATTAAAA
GST-SaDnaD1-195-XhoI-C	GGGCTCGAGTTAAGAATCGTCAATTGT
GST-SaDnaDY176A-N	AGCTTTAAAGCTATGGATCGTATTTTA
GST-SaDnaDY176A-C	ACGATCCATAGCTTTAAAGCTAAGTTT

These plasmids were verified by DNA sequencing. Underlined nucleotides indicate the designated site for the restriction site or the mutation site.

*E*. *coli* BL21(DE3) cells were transformed with the expression vector, and overexpression of the proteins was induced through incubation with 1 mM of isopropyl thiogalactopyranoside for 8 h at 25°C. The cells were lysed in a GST loading buffer (140 mM NaCl, 2.7 mM KCl, 10 mM Na_2_HPO_4_, and 1.8 mM KH_2_PO4, pH 7.3). The GST fusion proteins GST-SaDnaD, GST-SaDnaDY176A, and GST-SaDnaD1-195 were purified from the soluble supernatant by GSTrap HP affinity column (GE Healthcare Bio-Sciences, Piscataway, NJ, USA) and were eluted with a GST elution buffer (50 mM Tris-HCl and 10 mM reduced glutathione, pH 8.0). The GST fusion proteins were cleaved by Factor Xa (25 μg/mL; Sigma—Aldrich, St. Louis, MO, USA) for 8 h at 25°C to remove the GST tag. After dialysis against Buffer C (20 mM potassium phosphate and 100mM NaCl, pH 7.0), the protein solution was applied to the Heparin HP column (GE Healthcare Bio-Sciences, Piscataway, NJ, USA) and was eluted with a linear NaCl gradient from 0.1 M to 0.7 M with Buffer C using the AKTA-FPLC system. After the analysis of SDS-PAGE, the peak fractions were collected and concentrated. For further purification and buffer exchange, the resultant protein solution was applied to a Superdex 200 HR 10/30 column equilibrated with Buffer B and purified by the AKTA-FPLC system. The peak fraction for tag-free protein (SaDnaD, SaDnaDY176A, and SaDnaD1-195) was collected and concentrated. Protein purity remained at >97% as determined by SDS-PAGE.

## Results

### Purification of SaDnaD

The gene *SAAV1440* encoding for the putative DnaD was PCR-amplified using the genomic DNA of *S*. *aureus* subsp. *aureus* ED98 as template. This amplified gene was then ligated into the pET21e vector for protein expression. SaDnaD was heterologously overexpressed in *E*. *coli* and then purified from the soluble supernatant by Ni^2+^-affinity chromatography (S1 Fig). Pure protein was obtained in this single chromatographic step with an elution of Buffer B and dialyzed against a dialysis buffer (Buffer C). Approximately >10 mg of purified protein was obtained from 1 L of *E*. *coli* cell culture. The truncated SaDnaD proteins were also purified according to the same protocol as that for the wild-type protein, and the purification results were similar.

### EMSA of SaDnaD

**Fig 1:**
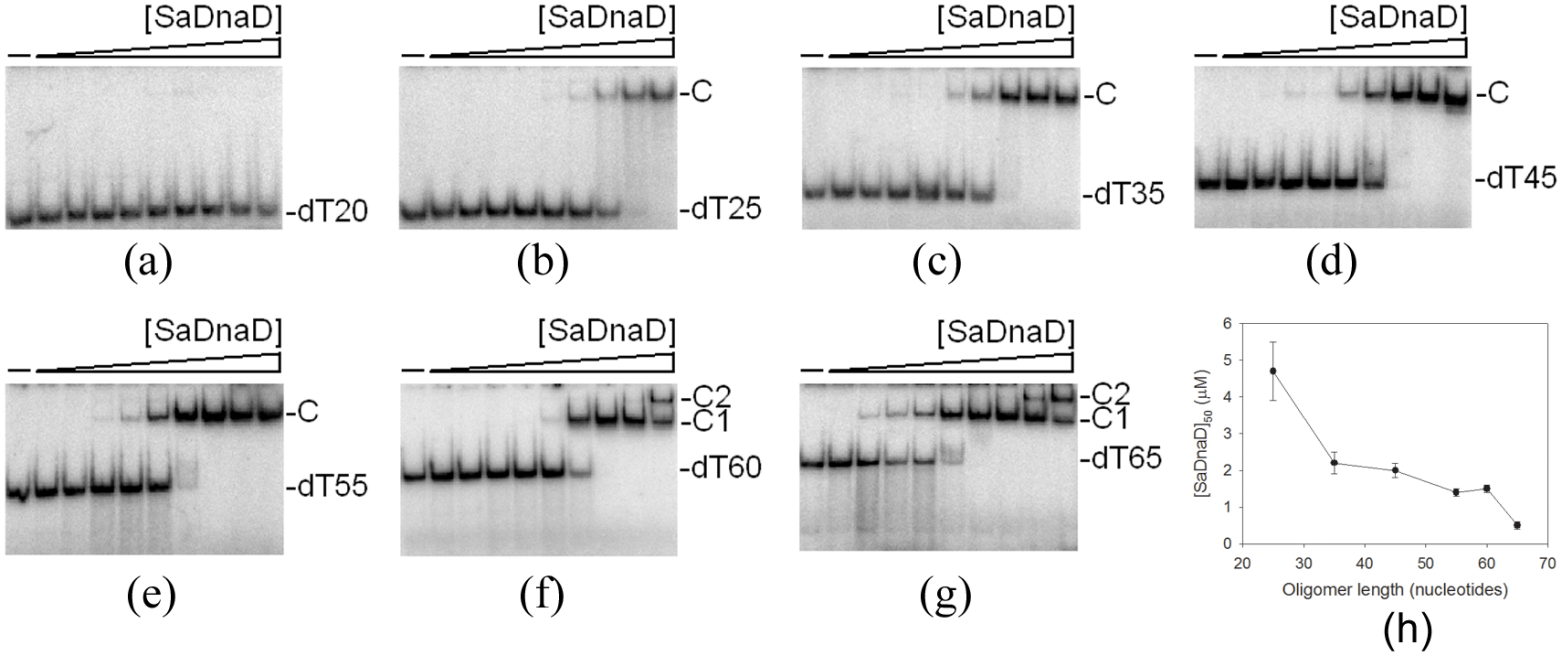
EMSA of SaDnaD. Protein (0, 0.06, 0.12, 0.25, 0.5, 1, 2, 4, 8, and 16 μM; monomer) was incubated at 25°C for 30 min with 1.7 nM of (A) dT20, (B) dT25, (C) dT35, (D) dT45, (E) dT55, (F) dT60, or (G) dT65 in a total volume of 10 μL in 20 mM Tris—HCl (pH 8.0) and 100 mM NaCl. The resulting samples were mixed with gel-loading solution (0.25% bromophenol blue and 40% sucrose; w/v), resolved on a native 8% polyacrylamide gel (8.3 × 7.3 cm) at 4°C in TBE buffer for 1–1.5 h at 100 V, and were visualized by phosphorimaging. The phosphor storage plate was scanned, and the data for complex and free DNA bands were digitized for quantitative analysis. (H) Summary of the [SaDnaD]_50_ values of SaDnaD. The [SaDnaD]_50_ values of SaDnaD as a function of the length of the ssDNA determined using EMSA.

To investigate the length of nucleotides needed for the formation of the DnaD—ssDNA complex, as well as the ssDNA-binding ability of DnaD, we studied the binding of SaDnaD to dT20, dT25, dT35, dT45, dT55, dT60, and dT65 (Fig 1) with different protein concentrations using EMSA. When we incubated SaDnaD with dT20, no significant band shift was observed. This result indicates that SaDnaD could not form a stable complex with this homopolymer during electrophoresis (Fig 1A). Some smears were observed; thus, SaDnaD appeared to interact with dT20, but the ssDNA may be too short to form a stable complex with SaDnaD. In contrast to dT20, longer dT homopolymers, such as dT25–55 (Fig 1B–1E), produced a significant band shift (C, complex). These findings indicate that the ssDNA-binding activity of SaDnaD is strong enough to form a stable protein—DNA complex in solution. Two different complexes for dT60 (Fig 1F) and dT65 (Fig 1G) were formed by SaDnaD.

### Binding constants of SaDnaD—ssDNA complexes determined from EMSA

Binding of SaDnaD to ssDNA of different lengths formed distinct complexes. To compare the binding abilities of SaDnaD to ssDNA of different lengths, the midpoint values for input ssDNA binding that were calculated from the titration curves of EMSA and the [Protein]_50_ were quantified using linear interpolation from the protein concentration and are summarized in Table 3. As shown in Fig 1H, the DNA binding abilities of SaDnaD were length-dependent. The [SaDnaD]_50_ for dT65 binding is 0.5 ± 0.1 μM, which is ninefold lower than that for dT25 binding (4.7 ± 0.8 μM). Thus, the protein—DNA contact for each complex of SaDnaD may not be similar.

**Table 3:** The [SaDnaD]_50_ values of SaDnaD as analyzed by EMSA.

DNA	[Protein]_50_ (μM)
dT20	ND
dT25	4.7 ± 0.8
dT35	2.2 ± 0.3
dT45	2.0 ± 0.2
dT55	1.4 ± 0.1
dT60	1.5 ± 0.1
dT65	0.5 ± 0.1

[Protein]_50_ was calculated from the titration curves of EMSA by determining the concentration of the protein (μM) needed to achieve the midpoint value for input DNA binding. For dT60 and dT65, input ssDNA binding was the sum of the intensities from the two separate ssDNA—protein complexes. Errors are standard deviations determined by three independent titration experiments.

### C-terminal region of SaDnaD is crucial for ssDNA binding

Given that many conserved positively charged residues are located in the C-terminal domain of DnaD [35], the truncated SaDnaD proteins, namely, SaDnaD1-204 (the C-terminal 24 amino acid residues were removed), SaDnaD1-200, and SaDnaD1-195, were constructed and purified (S1 Fig) to investigate whether the C-terminal region is essential for ssDNA binding and SaDnaD oligomerization. The binding of SaDnaD1-195, SaDnaD1-200, and SaDnaD1-204 to dT40 was examined using EMSA. No significant band shift was observed for SaDnaD1-195, SaDnaD1-200, and SaDnaD1-204 incubated with dT40, which suggests no complex formation (S2 Fig). Because some smears were observed, SaDnaD1-204 appeared to interact with dT40, but cannot form a stable complex with dT40 during electrophoresis. Thus, the C-terminal region of SaDnaD (the amino acid residues 205–228) is crucial for ssDNA binding.

### Oligomeric state of SaDnaD in solution

**Fig 2:**
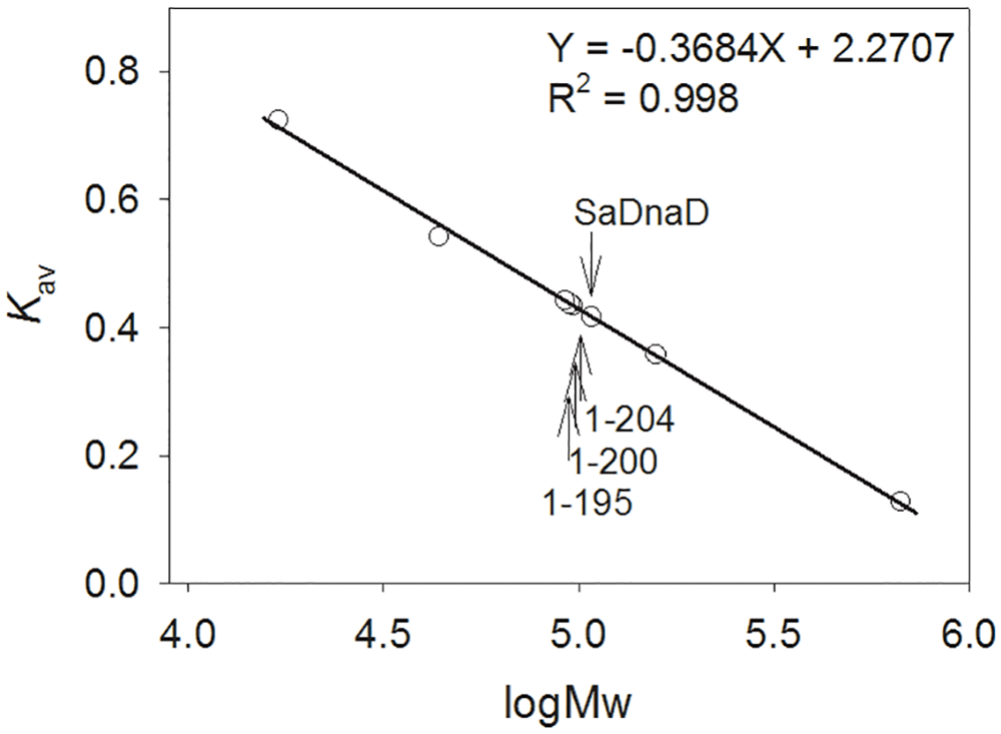
Gel-filtration chromatographic analysis. Gel-filtration chromatography was carried out by the AKTA-FPLC system. The column was calibrated with proteins of known molecular weight: thyroglobulin (670 kDa), γ-globulin (158 kDa), ovalbumin (44 kDa), and myoglobin (17 kDa). The *K*_av_ values for the standard proteins and the SaDnaD variants were calculated from the equation: *K*_av_ = (*V*_e_ − *V*_o_)/(*V*_c_ − *V*_o_), where *V*_o_ is the column void volume, *V*_e_ is the elution volume, and *V*_c_ is the geometric column volume. A standard linear regression curve was generated by plotting the log of the molecular mass of the calibration proteins against their *K*_av_ values.

The analysis of purified SaDnaD protein (4 mg/mL) by gel filtration chromatography revealed a single peak with elution volume of 74.3 mL. Assuming that the shape and partial specific volume of SaDnaD are similar to the standard proteins, the native molecular mass of SaDnaD was estimated to be 107895 Da, which was calculated from a standard linear regression equation, *K*_av_ = −0.3684 (logMw) + 2.2707 (Fig 2). The native molecular mass for SaDnaD is approximately four times the molecular mass of a SaDnaD monomer (~27 kDa). Thus, we conclude that SaDnaD in solution is a stable tetramer.

Purified SaDnaD1-195, SaDnaD1-200, and SaDnaD1-204 were also analyzed by gel filtration chromatography to investigate the deletion effect in the oligomeric state of SaDnaD. The chromatographic results showed a single peak with elution volumes of 75.6, 75.9, and 76.3 mL for SaDnaD1-204, SaDnaD1-200, and SaDnaD1-195, respectively. The native molecular masses of SaDnaD1-204, SaDnaD1-200, and SaDnaD1-195 were estimated to be 96942, 94872, and 92140 Da, respectively. Thus, SaDnaD1-204, SaDnaD1-200, and SaDnaD1-195 in solution are stable tetramers, which were similar to SaDnaD. Although these deletion mutants of SaDnaD cannot bind to ssDNA (S2 Fig), deletion of the C-terminal region does not cause any change in the oligomeric state of SaDnaD, consistent with what was previously shown for the *B*. *subtilis* DnaD [27, 35].

### Fluorescence detection of SaDnaD and ssDNA interaction

**Fig 3:**
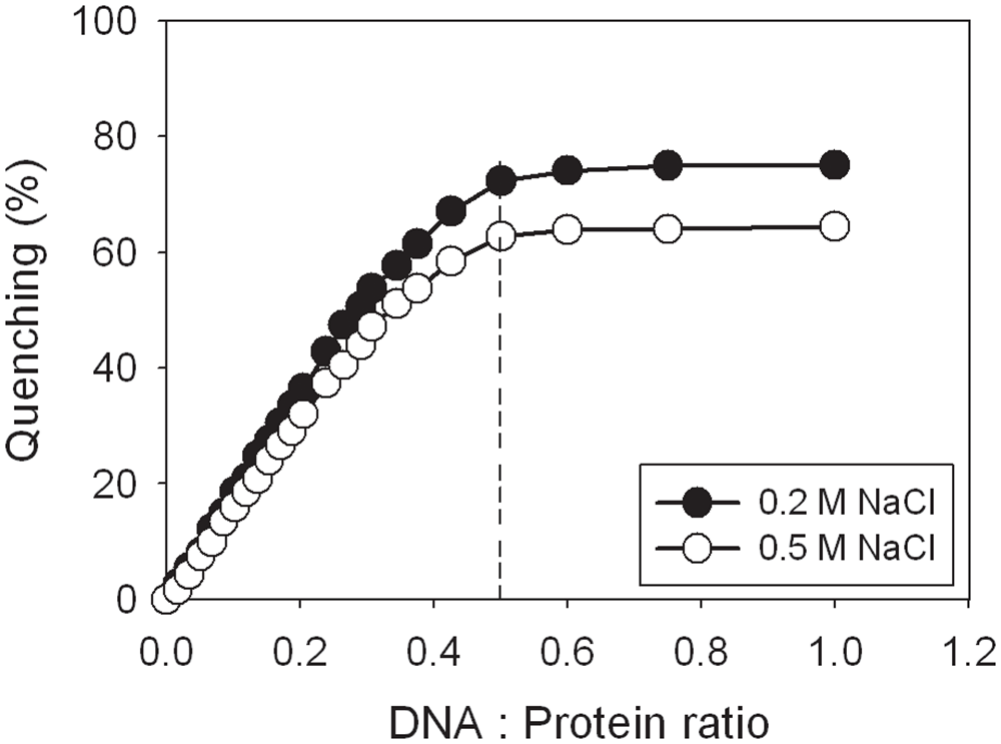
Fluorescence quenching. The excitation and emission of tryptophan fluorescence were detected at 295 and 340 nm, respectively. The protein solution (0.1 μM; tetramer) in 2 mL Tris—HCl buffer (20 mM Tris—HCl, and pH 8.0) containing 200 or 500 mM NaCl was titrated with increasing quantities of dT65 oligonucleotide. After the addition of ssDNA, the complex solution was equilibrated for 300 s until no fluorescence change could be observed.

We examined the quenching fluorescence intensity of tryptophan in SaDnaD upon addition of ssDNA. SaDnaD has three tryptophan residues, namely, Trp148, Trp184, and Trp219, allowing ssDNA binding analysis through tryptophan fluorescence quenching. The protein displayed strong intrinsic fluorescence with a peak wavelength of 340 nm when excited at 295 nm, which is consistent with tryptophan fluorescence. Upon the addition of ssDNA, fluorescence quenching of SaDnaD was observed. The intrinsic fluorescence was progressively quenched as dT65 was titrated into the SaDnaD solution, suggesting an interaction of SaDnaD with dT65 (Fig 3). Upon adding a saturating quantity of ssDNA in the presence of 200 and 500 mM NaCl, the intrinsic fluorescence at 340 nm was quenched by 75% and 65%, respectively. Using these fluorescence data, we calculated the binding stoichiometry between SaDnaD and ssDNA. The saturation curves of SaDnaD in the presence of 200 and 500 mM NaCl suggest that the binding is stoichiometric at 0.5 SaDnaD tetramer per 65-mer ssDNA if enough binding sites were present in the DNA for all SaDnaD molecules to bind. Therefore, the binding-site size of SaDnaD tetramer estimated from fluorescence quenching is approximately 32 nt (65 × 0.5 = 32.5). We also tested the fluorescence quenching of SaDnaD1-195, SaDnaD1-200, and SaDnaD1-204 by adding ssDNA. The ssDNA dT65 at the DNA—protein ratio of 1 only quenched <5%, <10%, and ~20% of SaDnaD1-195, SaDnaD1-200, and SaDnaD1-204 fluorescence intensities, respectively. Thus, the ssDNA-binding abilities of SaDnaD1-195, SaDnaD1-200, and SaDnaD1-204 were significantly impaired and their ssDNA-binding site sizes were not estimated.

### Characterization of SaDnaD bound to SaPriA or SaDnaA

**Fig 4:**
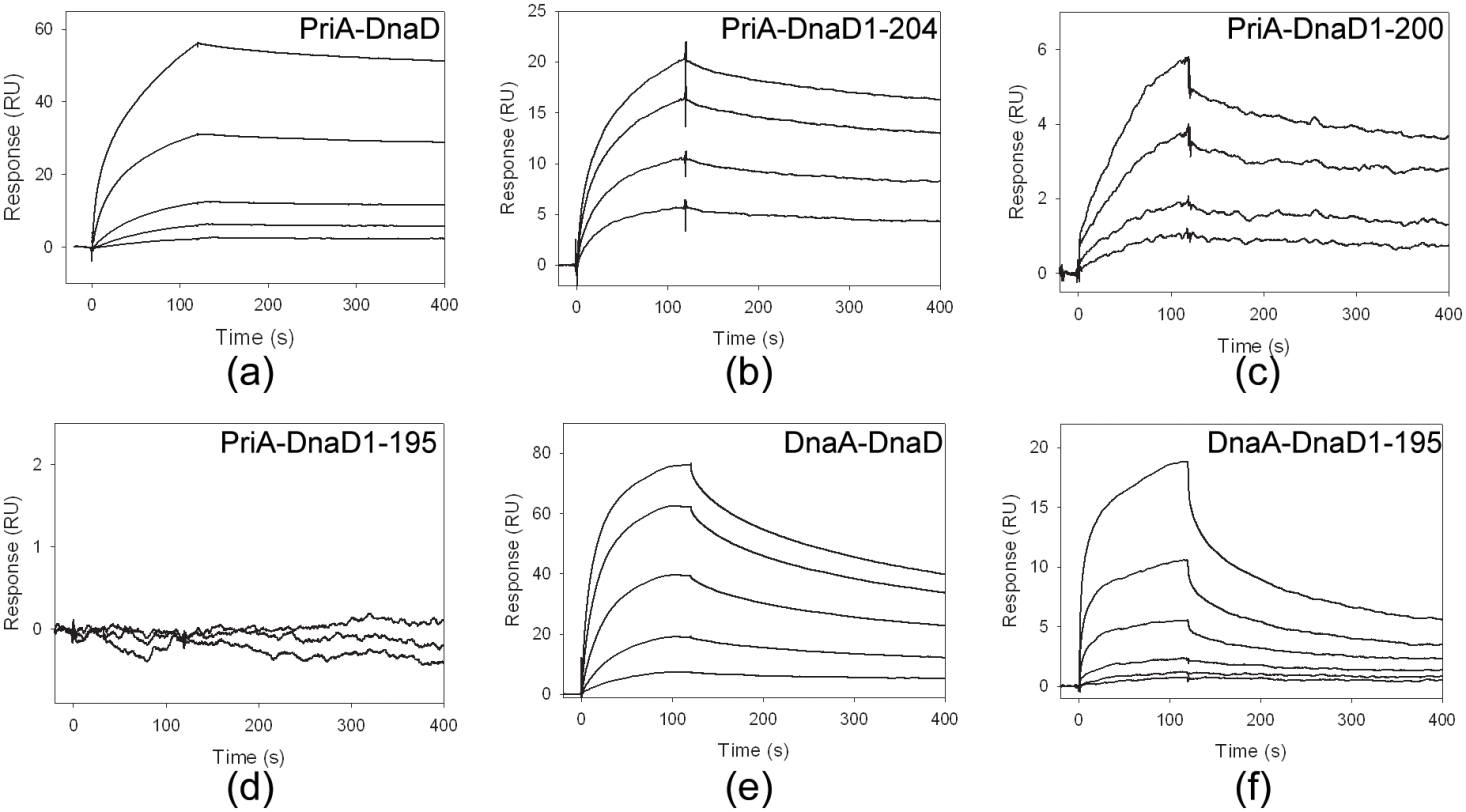
SPR analysis. Protein—protein interactions within (A) PriA—DnaD, (B) PriA—DnaD1-204, (C) PriA—DnaD1-200, (D) PriA—DnaD1-195, (E) DnaA—DnaD and (F) DnaA—DnaD1-195 were analyzed using SPR. SaPriA and SaDnaA were individually immobilized on Series S sensor chips CM5, and the binding experiments were carried out using a Biacore T200 (GE Healthcare Bio-Sciences, Piscataway, NJ, USA). Control samples were used to monitor the sensor chip surface stability, demonstrating reproducibility throughout the duration of the experiments. The estimated *K*_d_ values were derived by fitting the association and dissociation signals with a 1:1 (Langmuir) model using the Biacore T200 Evaluation Software. The residual plots for SPR fitting were shown in S3 Fig.

*B*. *subtilis* DnaD—DnaA interaction is previously identified through the yeast two-hybrid analysis [26]. To gain insights into direct interactions and further quantitatively characterize whether the C-terminal domain of DnaD was involved in protein—protein interactions within PriA—DnaD or DnaA—DnaD, we provided experimental evidence using SPR to investigate the effect of various fragments on the ability of DnaD to bind to PriA or DnaA. PriA and DnaA were individually immobilized on a sensor chip (as a ligand), and the DnaD solution (as an analyte) was passed over the sensor chip in a microfluidic chamber. Fig 4 shows the SPR results at various DnaD concentrations. The *K*_d_ values were calculated from the equilibrium binding isotherms using a simple binding model (a 1:1 Langmuir binding model). As shown in Table 4, C-terminal deletion of DnaD resulted in decreased *K*_d_ values for PriA and DnaA binding, indicating a crucial role for protein—protein interactions. DnaD1-195 was injected at increasing concentrations, but the binding response for PriA did not change; this result indicates that aa 196–228 is necessary for PriA binding in DnaD (Fig 4D) but not for DnaA binding (Fig 4F). Therefore, DnaD may bind to PriA in a different manner from that to DnaA.

**Table 4:** Protein—protein interactions within PriA—DnaD, PriA—DnaD1-204, PriA—DnaD1-200, PriA—DnaD1-195, DnaA—DnaD, and DnaA—DnaD1-195.

Ligand	Analyte	*K*_d_ (M)
PriA	DnaD	2.0 ± 0.3 x 10^−9^
PriA	DnaD1-204	1.1 ± 0.2 x 10^−8^
PriA	DnaD1-200	2.6 ± 0.3 x 10^−8^
PriA	DnaD1-195	ND
DnaA	DnaD	1.7 ± 0.2 x 10^−8^
DnaA	DnaD1-195	1.5 ± 0.2 x 10^−7^

The estimated *K*_d_ values were derived by fitting the association and dissociation signals with a 1:1 (Langmuir) model using the Biacore T200 Evaluation Software.

**Fig 5:**
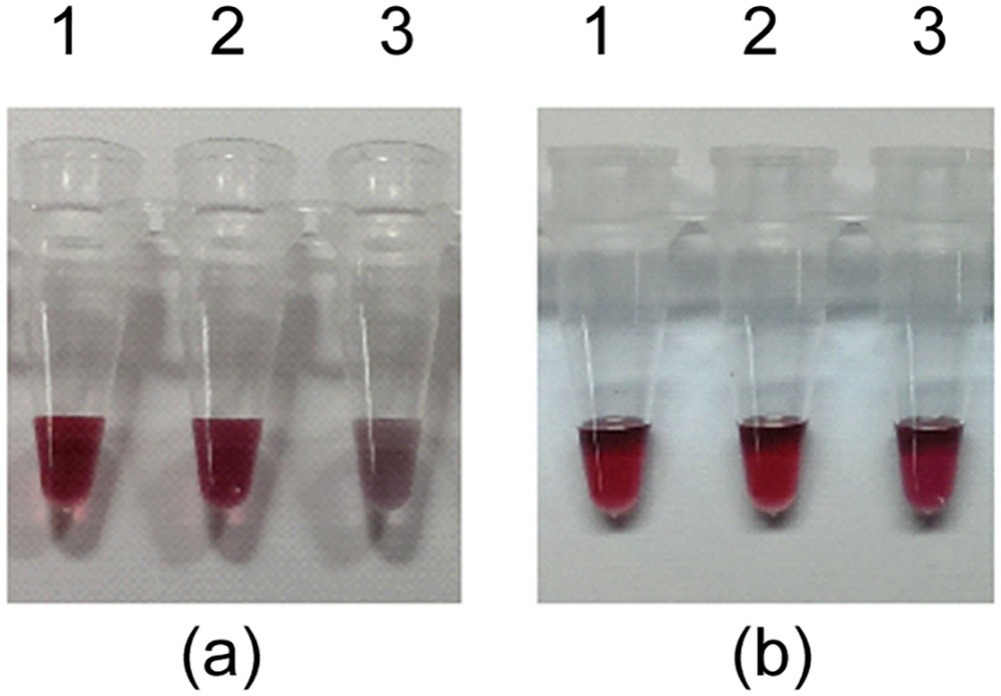
Gold nanoparticle assay. (A) Analysis of SaDnaA—SaDnaD1-195 interaction using SaDnaA (lane 1), SaDnaD1-195 (lane 2), and mixture of SaDnaA and SaDnaD1-195 (lane 3). (B) Analysis of SaPriA-SaDnaD1-195 interaction using SaPriA (lane 1), SaDnaD1-195 (lane 2), and mixture of SaPriA and SaDnaD1-195 (lane 3). The gold nanoparticle assay showed that the solution of SaDnaA—SaDnaD1-195 changed from red to purple, whereas that of SaPriA-SaDnaD1-195 maintained the red color

To rapidly confirm whether SaDnaD1-195 can interact with SaDnaA and SaPriA, we carried out gold nanoparticle assays. As histidine-tagged proteins, SaDnaD1-195, SaPriA, and SaDnaA can be immobilized on commercialized Ni^2+^-NTA SAM nanoparticles. Protein-loaded nanoparticles retain the same red color as the unmodified SAM-coated nanoparticles. However, the solution turns purple when gold nanoparticles aggregate, indicating the protein—protein interactions (Fig 5). The gold nanoparticle assay showed that the solution of SaDnaA—SaDnaD1-195 changed from red to purple (Fig 5A), whereas that of SaPriA-SaDnaD1-195 maintained the red color (Fig 5B). Thus, the C-terminal region aa 196–228 in SaDnaD is relatively important for SaPriA binding, which is consistent with the SPR results.

### SaDnaD can significantly stimulate the ATPase activity of SaPriA but SaDnaD1-195 cannot

Gram-negative bacterial PriA is known as a poor helicase when acting alone in vitro [45]. *E*. *coli* PriA activity can be significantly stimulated by PriB and SSB [46,47,48]. Whether SaDnaD and SaDnaD1-195 can enhance SaPriA ATPase is unknown. SaPriA could hydrolyze ATP alone, and this ATPase activity was dramatically stimulated (twelvefold) in the presence of SaDnaD (S4 Fig). However, when acting with SaDnaD1-195, the stimulation of SaPriA ATPase was insignificant (only twofold). Thus, the C-terminal region of SaDnaD is important for the stimulation of SaPriA ATPase activity.

### SaDnaDY176A significantly stimulated the ATPase activity of SaPriA as SaDnaD did

In this study, we determined that the ATPase activity of PriA can be significantly stimulated via contact with the C-terminal region of DnaD (Fig 4). We further investigated whether the DNA binding ability of DnaD is related to the stimulation effect. The highly conserved DNA-binding motif of DnaD, YxxxIxxxW, has been identified [35]. The Y180A mutant protein of *B*. *subtilis* DnaD cannot bind DNA [35]. The same DNA-binding motif is also found in SaDnaD, and the residue Y180 in *B*. *subtilis* DnaD is Y176 in SaDnaD. To assess whether the DNA binding activity of DnaD is also involved in the stimulation of the ATPase activity of PriA, tag-free SaDnaDY176A mutant protein was produced using GST fusion and Factor Xa (S5 Fig). To exclude the possible effect of a His tag, tag-free SaDnaD and SaDnaD1-195 were also constructed and used in the ATPase assay for analysis. The stimulation effect of a tag-free SaDnaD protein was also analyzed according to the same protocol as that for the His-tagged SaDnaD protein, and their results were similar (S6 Fig). SaPriA ATPase activity was dramatically stimulated (elevenfold to twelvefold) in the presence of SaDnaD. However, when acting with SaDnaD1-195, the stimulation of SaPriA ATPase was insignificant (only twofold). We also used tag-free SaDnaDY176A mutant protein for this analysis and obtained a similar result to that of the wild-type protein. Therefore, the DNA-binding ability of DnaD and the His tag fused in the C terminus of DnaD did not play a role in the stimulation of SaPriA ATPase activity. However, SaDnaD1-195 still partially stimulated the PriA ATPase activity (S6 Fig). How SaDnaD1-195 can slightly enhance the ATPase activity of SaPriA remains unclear.

### Effect of SaDnaD, SaDnaD1-195, and SaDnaDY176A on the kinetic parameters of SaPriA ATPase

**Fig 6:**
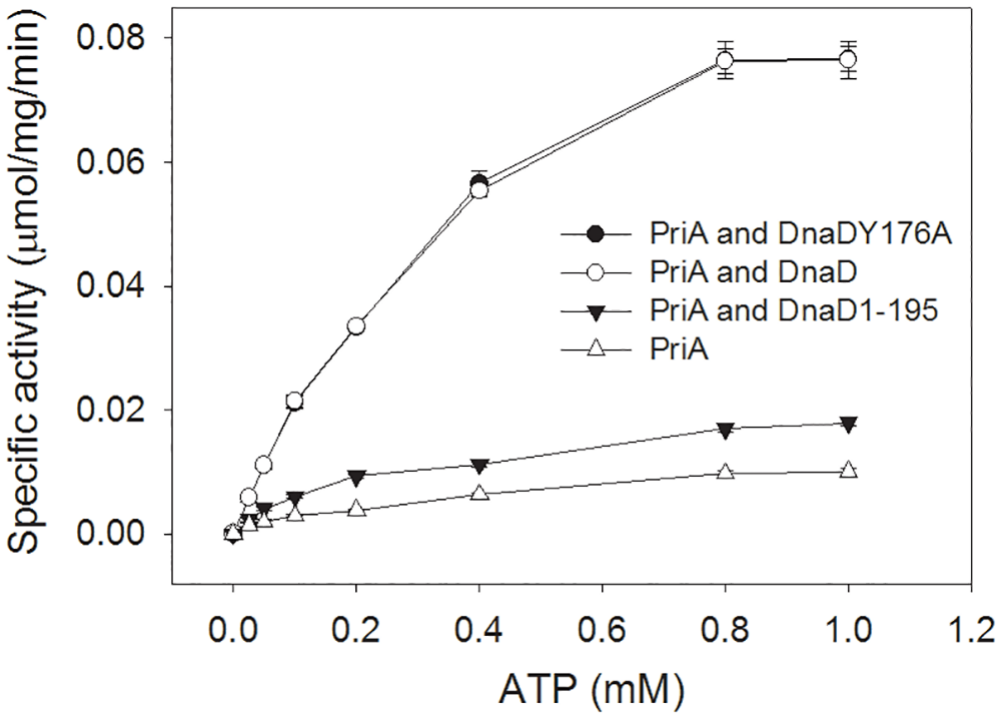
Effect of SaDnaD, SaDnaD1-195, and SaDnaDY176A on the kinetic parameters of the SaPriA ATPase. In this experiment, tag-free SaDnaD proteins were used for analysis of SaPriA ATPase activity. SaPriA ATPase assay was performed with 0–1 mM [γ-32P] ATP, 0.125 μM of SaPriA, and 0.1 μM PS4/PS3-dT25 DNA substrate for 1 h. To study the effect, SaDnaD (10 μM), SaDnaDY176A (10 μM), or SaDnaD1-195 (10 μM) was added into the assay solution. Aliquots (5 μL) were taken and spotted onto a polyethyleneimine cellulose thin-layer chromatography plate, which was subsequently developed in 0.5 M formic acid and 0.25 M LiCl for 30 m. Reaction products were visualized by autoradiography and quantified with a Phosphorimager. Data points are an average of three determinations and the errors are standard deviation.

We further determined the kinetic parameters of PriA ATPase in the presence or absence of DnaD. Given that DnaD can bind and may stabilize the dsDNA substrate, the SaDnaDY176A mutant protein was also used for this analysis to rule out any possible role of the DNA-binding involvement of DnaD. As shown in Fig 6, the ATP hydrolyzing reaction was insignificant when only PriA was added into the assay mixture. Under the same ATP concentration assay range, the kinetic parameters of SaPriA ATPase varied when SaDnaD, SaDnaD1-195, and SaDnaDY176A protein was individually added in the hydrolyzing reaction (Table 5). When acting with SaDnaD or SaDnaDY176A, the *V*_max_ values of SaPriA ATPase increased fivefold, whereas the *K*_m_ values were only slightly affected. The addition of SaDnaD1-195 to the hydrolyzing reaction induced only a minimal effect.

**Table 5:** Apparent Michaelis—Menten constants for SaPriA in the presence of the SaDnaD protein.

	PriA			
DnaD	*V*_max_	*K*_m_	*V*_max_/*K*_m_	Fold
PriA	0.023 ± 0.001	0.47 ± 0.07	0.049	1.0
PriA and DnaD	0.114 ± 0.005	0.44 ± 0.04	0.259	5.3
PriA and DnaD1-195	0.026 ± 0.003	0.42 ± 0.09	0.062	1.2
PriA and DnaDY176A	0.115 ± 0.005	0.46 ± 0.04	0.250	5.1

The kinetic parameters *K*m (mM) and *V*max (μmol/mg/min) were determined by fitting the hydrolyzing rate from individual experiments to the Michaelis—Menten equation (Enzyme Kinetics module of Sigma-Plot; Systat Software, Chicago, IL, USA), and then the standard errors were given. The enzyme activity of PriA for kinetic fitting is an average determined at three measurements.

## Discussion

*S*. *aureus* is a Gram-positive pathogen that exhibits a remarkable ability to develop antibiotic resistance [49,50,51,52,53,54]. Considering that PriA and DnaA-directed primosomes are required for bacterial DNA replication restart processes, these primosomes may be suitable targets for antibiotic development. Because PriA, DnaA, and DnaD are not found in mammals, inhibitors based on these proteins are potentially safe for human use. We found that SaDnaD1-195 can bind to DnaA and can form a tetramer, but it cannot interact with ssDNA and PriA. This binding information on SaDnaD is a prerequisite for new antibacterial drug development and for formulating any model of protein function in DNA replication and DNA replication restart.

In this study, we discovered that the primosomal DnaD protein, which is only found in the Gram-positive bacteria, can bind PriA helicase via the C-terminal region, and this interaction significantly stimulated the activity of PriA. PriA activity can also be stimulated by the Gram-negative *E*. *coli* PriB and SSB proteins [47,48]. For SSB, the 15 C-terminal amino acids are absolutely needed for PriA binding and stimulation. Moreover, the stimulation of PriA by the SSB C-terminus requires binding of SSB to ssDNA within the substrate [48]. For PriB, the stimulation of PriA by PriB needs to form a stable PriA-PriB-DNA complex. In addition, the ssDNA-containing DNA substrate is required to simulate PriA by PriB [47]. These two stimulatory cases of PriA by PriB and SSB indicate that both protein—protein interaction and DNA-binding ability are important factors in simulating PriA. However, several lines of evidence indicate that the stimulatory mechanism of PriA by DnaD, PriB or SSB may differ. First, unlike PriB and SSB, DnaD is not an OB-fold protein, thus binding ssDNA differently [38,55]. Second, DnaD is found to bind dsDNA (but with lower affinity than ssDNA) [35], whereas PriB and SSB are not. Third, the DNA-binding mutant SaDnaDY176A still stimulated PriA activity as the wild-type SaDnaD did, whereas the DNA-binding mutants of PriB and SSB do not exhibit a stimulation effect for PriA. Fourth, although the C-terminal region of DnaD was also crucial for the PriA stimulation as the case in SSB, the amino acid residues in SSB and DnaD C-terminal region are quite different and the sequences are not similar. Fifth, the C-terminal region of DnaD is important both for DNA- and PriA-binding, whereas the ssDNA binding region is located at the N-terminal domain of SSB, not the C terminus [55]. Occupying of partially unwound intermediates of DNA unwinding reactions by SSB is known to prevent ssDNA reannealing and enhance the apparent processivity of more distributive helicases. If the DNA binding activity of DnaD plays no role in the activity stimulation of PriA, how can the protein—protein interactions within PriA-DnaD can conduct such effect? The results from our kinetic studies further reveal that PriA binding to DnaD did not significantly change the *K*_m_ value. Thus, DnaD binding to PriA may not alter the ATP binding environment of PriA. Whether the conformation of PriA changes upon DnaD binding and whether the increased *V*_max_ value of PriA ATPase for DnaD binding results from facilitating the product (ADP) release remains undiscovered.

Roles of the N-terminal domain of DnaD have been well established. The structure and detailed functions of the C-terminal domain of DnaD are still poorly understood. The C-terminal region of DnaD was found to be crucial for both PriA and ssDNA binding, suggesting that these binding sites are overlapped on DnaD. Future studies should focus on whether the binding site for ssDNA on DnaD is necessary to overlap the binding site for PriA, because PriA (a monomer) and DnaD (a tetramer) have different oligomerization states. For example, only one monomer of the PriB dimer can interact with the DNA and its partner proteins [38,56]. The complex structures of DnaD are useful in helping us to understand the primosome assembly mechanisms.

Most DNA helicases of superfamily I and superfamily II are almost non-hexameric and have poor dsDNA unwinding activities when acting alone in vitro [45]. *E*. *coli* PriA helicase activity can be significantly stimulated by PriB and SSB. Determining whether DnaD can enhance the Gram-positive PriA helicase or the Gram-negative PriA helicase is important to understand deeply the role of DnaD in the primosome assembly. SPR results indicate that the binding sites on DnaD for PriA and ssDNA do not overlap with the binding site for DnaA (Fig 4), suggesting that the DnaD expression in cells may simultaneously work with DnaA in replication and with PriA in replication restart.

## Supporting Information

S1 Fig Protein purity. Coomassie Blue-stained SDS-PAGE (12%) of the purified SaDnaD (lane 1), SaDnaD1-204 (lane 2), SaDnaD1-200 (lane 3), SaDnaD1-195 (lane 4) and molecular mass standards (M) are shown. The sizes of the standard proteins, from the top down, are as follows: 170, 130, 100, 70, 55, 40, 35, 25, 15, and 10 kDa.
(TIF)

S2 Fig ssDNA binding analysis of the deletion mutants of SaDnaD. (A) SaDnaD1-195, (B) SaDnaD1-200, or (C) SaDnaD1-204, was incubated at 25°C for 30 min with 1.7 nM dT40 in a total volume of 10 μL in 20 mM Tris—HCl (pH 8.0) and 100 mM NaCl. The resulting samples were mixed with gel-loading solution (0.25% bromophenol blue and 40% sucrose; w/v), resolved on a native 8% polyacrylamide gel (8.3 × 7.3 cm) at 4°C in TBE buffer for 1–1.5 h at 100 V, and were visualized by phosphorimaging. The phosphor storage plate was scanned, and the data for complex and free DNA bands were digitized for quantitative analysis.
(TIF)

S3 Fig Residual plots for SPR fitting. The estimated *K*_d_ values were derived by fitting the association and dissociation signals with a 1:1 (Langmuir) model using the Biacore T200 Evaluation Software. These residual plots were the calculated difference between the experimental and fitted data [57] for PriA—DnaD, PriA—DnaD1-204, PriA-DnaD1-200, DnaA—DnaD, and DnaA—DnaD1-195, respectively.
(TIF)

S4 Fig SaDnaD can significantly stimulate the ATPase activity of SaPriA but SaDnaD1-195 cannot. SaPriA ATPase assay was performed with 0.4 mM [γ-32P] ATP, 0.125 μM of SaPriA, and 0.1 μM PS4/PS3-dT25 DNA substrate for 1 h. To study the effect, SaDnaD (10 μM) or SaDnaD1-195 (10 μM) was added into the assay solution. Aliquots (5 μL) were taken and spotted onto a polyethyleneimine cellulose thin-layer chromatography plate, which was subsequently developed in 0.5 M formic acid and 0.25 M LiCl for 30 m. Reaction products were visualized by autoradiography and quantified with a Phosphorimager.
(TIF)

S5 Fig Production of tag-free SaDnaD proteins. To obtain proteins without a His tag, tag-free SaDnaD, SaDnaDY176A, and SaDnaD1-195 proteins were produced using GST fusion and Factor Xa and were used in succeeding analysis. Coomassie Blue-stained SDS-PAGE (12%) of the purified SaDnaD (lane 1), GST-SaDnaD (lane 2), SaDnaD1-195 (lane 3), GST-SaDnaD1-195 (lane 4), GST-SaDnaDY176A (lane 5), SaDnaDY176A (lane 6), and molecular mass standards (M) are shown. The sizes of the standard proteins, from the top down, are as follows: 170, 130, 100, 70, 55, 40, 35, 25, and 15 kDa.
(TIF)

S6 Fig SaDnaDY176A significantly stimulate the ATPase activity of SaPriA as SaDnaD did. In this experiment, tag-free SaDnaD proteins were used for analysis of SaPriA ATPase activity. SaPriA ATPase assay was performed with 0.4 mM [γ-32P] ATP, 0.125 μM of SaPriA, and 0.1 μM PS4/PS3-dT25 DNA substrate for 1 h. To study the effect, SaDnaD (10 μM), SaDnaDY176A (10 μM), or SaDnaD1-195 (10 μM) was added into the assay solution. Aliquots (5 μL) were taken and spotted onto a polyethyleneimine cellulose thin-layer chromatography plate, which was subsequently developed in 0.5 M formic acid and 0.25 M LiCl for 30 m. Reaction products were visualized by autoradiography and quantified with a Phosphorimager.
(TIF)

## References

[1] Merrikh H, Machon C, Grainger WH, Grossman AD, Soultanas P (2011) Co-directional replication-transcription conflicts lead to replication restart. Nature 470: 554–557. 10.1038/nature09758 21350489 PMC3059490

[2] McHenry CS (2011) DNA replicases from a bacterial perspective. Annu. Rev. Biochem. 80: 403–436. 10.1146/annurev-biochem-061208-091655 21675919

[3] Leonard AC, Grimwade JE (2011) Regulation of DnaA assembly and activity: taking directions from the genome. Annu. Rev. Microbiol. 65: 19–35. 10.1146/annurev-micro-090110-102934 21639790 PMC4075013

[4] Mott ML, Berger JM (2007) DNA replication initiation: mechanisms and regulation in bacteria. Nat. Rev. Microbiol. 5: 343–354. 17435790 10.1038/nrmicro1640

[5] Heller RC, Marians KJ (2006) Replisome assembly and the direct restart of stalled replication forks. Nat. Rev. Mol. Cell. Biol. 7: 932–943. 17139333 10.1038/nrm2058

[6] McGlynn P, Lloyd RG (2002) Recombinational repair and restart of damaged replication forks. Nat. Rev. Mol. Cell. Biol. 3: 859–870. 12415303 10.1038/nrm951

[7] Cox MM (2001) Recombinational DNA repair of damaged replication forks in *Escherichia coli*: questions. Annu. Rev. Genet. 35: 53–82. 11700277 10.1146/annurev.genet.35.102401.090016

[8] Cox MM, Goodman MF, Kreuzer KN, Sherratt DJ, Sandler SJ, Marians KJ (2000) The importance of repairing stalled replication forks. Nature 404: 37–41. 10716434 10.1038/35003501

[9] Sandler SJ (2000) Multiple genetic pathways for restarting DNA replication forks in *Escherichia coli* K-12. Genetics 155: 487–497. 10835375 10.1093/genetics/155.2.487 PMC1461104

[10] Marians KJ (2000) PriA-directed replication fork restart in *Escherichia coli*. Trends Biochem. Sci. 25: 185–189. 10754552 10.1016/s0968-0004(00)01565-6

[11] Schekman R, Weiner A, Kornberg A (1974) Multienzyme systems of DNA replication. Science 186: 987–993. 4620044 10.1126/science.186.4168.987

[12] Masai H, Tanaka T, Kohda D (2010) Stalled replication forks: making ends meet for recognition and stabilization. Bioessays 32: 687–697. 10.1002/bies.200900196 20658707

[13] Tanaka T, Mizukoshi T, Sasaki K, Kohda D, Masai H (2007) *Escherichia coli* PriA protein, two modes of DNA binding and activation of ATP hydrolysis. J. Biol. Chem. 282: 19917–19927. 17483094 10.1074/jbc.M701848200

[14] Sasaki K, Ose T, Okamoto N, Maenaka K, Tanaka T, Masai H, et al (2007) Structural basis of the 3'-end recognition of a leading strand in stalled replication forks by PriA. EMBO J. 26: 2584–2593. 17464287 10.1038/sj.emboj.7601697 PMC1868909

[15] Mizukoshi T, Tanaka T, Arai K, Kohda D, Masai H (2003) A critical role of the 3' terminus of nascent DNA chains in recognition of stalled replication forks. J. Biol. Chem. 278: 42234–42239. 12917421 10.1074/jbc.C300285200

[16] Tanaka T, Mizukoshi T, Taniyama C, Kohda D, Arai K, Masai H (2002) DNA binding of PriA protein requires cooperation of the N-terminal D-loop/arrested-fork binding and C-terminal helicase domains. J. Biol. Chem. 277: 38062–38071. 12151393 10.1074/jbc.M204397200

[17] Briggs GS, Smits WK, Soultanas P (2012) Chromosomal replication initiation machinery of low-G+C-content *Firmicutes*. J. Bacteriol. 194: 5162–5170. 10.1128/JB.00865-12 22797751 PMC3457243

[18] Soultanas P (2012) Loading mechanisms of ring helicases at replication origins. Mol. Microbiol. 84: 6–16. 10.1111/j.1365-2958.2012.08012.x 22417087 PMC3401641

[19] Bruand C, Ehrlich SD, Janniere L (1995) Primosome assembly site in *Bacillus subtilis*. EMBO J. 14: 2642–2650. 7781616 10.1002/j.1460-2075.1995.tb07262.x PMC398378

[20] Huang YH, Huang CY (2014) Structural insight into the DNA-binding mode of the primosomal proteins PriA, PriB, and DnaT. Biomed. Res. Int. 2014: 195162 10.1155/2014/195162 25136561 PMC4129139

[21] Bruand C, Velten M, McGovern S, Marsin S, Serena C, Ehrlich SD, et al (2005) Functional interplay between the *Bacillus subtilis* DnaD and DnaB proteins essential for initiation and re-initiation of DNA replication. Mol. Microbiol. 55: 1138–1150. 15686560 10.1111/j.1365-2958.2004.04451.x

[22] Turner IJ, Scott DJ, Allen S, Roberts CJ, Soultanas P (2004) The *Bacillus subtilis* DnaD protein: a putative link between DNA remodeling and initiation of DNA replication. FEBS Lett. 577: 460–464. 15556628 10.1016/j.febslet.2004.10.051 PMC3033577

[23] Soultanas P (2002) A functional interaction between the putative primosomal protein DnaI and the main replicative DNA helicase DnaB in *Bacillus*. Nucleic Acids Res. 30: 966–974. 11842108 10.1093/nar/30.4.966 PMC100333

[24] Marsin S, McGovern S, Ehrlich SD, Bruand C, Polard P (2001) Early steps of *Bacillus subtilis* primosome assembly. J. Biol. Chem. 276: 45818–45825. 11585815 10.1074/jbc.M101996200

[25] Bruand C, Farache M, McGovern S, Ehrlich SD, Polard P (2001) DnaB, DnaD and DnaI proteins are components of the *Bacillus subtilis* replication restart primosome. Mol. Microbiol. 42: 245–255. 11679082 10.1046/j.1365-2958.2001.02631.x

[26] Ishigo-Oka D, Ogasawara N, Moriya S (2001) DnaD protein of *Bacillus subtilis* interacts with DnaA, the initiator protein of replication. J. Bacteriol. 183: 2148–2150. 11222620 10.1128/JB.183.6.2148-2150.2001 PMC95117

[27] Carneiro MJ, Zhang W, Ioannou C, Scott DJ, Allen S, Roberts CJ, et al (2006) The DNA-remodelling activity of DnaD is the sum of oligomerization and DNA-binding activities on separate domains. Mol. Microbiol. 60: 917–924. 16677303 10.1111/j.1365-2958.2006.05152.x PMC3035175

[28] Smits WK, Goranov AI, Grossman AD (2010) Ordered association of helicase loader proteins with the *Bacillus subtilis* origin of replication in vivo. Mol. Microbiol. 75: 452–461. 10.1111/j.1365-2958.2009.06999.x 19968790 PMC2992960

[29] Bonilla CY, Grossman AD (2012) The primosomal protein DnaD inhibits cooperative DNA binding by the replication initiator DnaA in *Bacillus subtilis*. J. Bacteriol. 194: 5110–5117. 10.1128/JB.00958-12 22821970 PMC3430336

[30] Zhang W, Machon C, Orta A, Phillips N, Roberts CJ, Allen S, et al (2008) Single-molecule atomic force spectroscopy reveals that DnaD forms scaffolds and enhances duplex melting. J. Mol. Biol. 377: 706–714. 10.1016/j.jmb.2008.01.067 18291414 PMC3033579

[31] Zhang W, Allen S, Roberts CJ, Soultanas P (2006) The *Bacillus subtilis* primosomal protein DnaD untwists supercoiled DNA. J. Bacteriol. 188: 5487–5493. 16855238 10.1128/JB.00339-06 PMC1540042

[32] Zhang W, Carneiro MJ, Turner IJ, Allen S, Roberts CJ, Soultanas P (2005) The *Bacillus subtilis* DnaD and DnaB proteins exhibit different DNA remodelling activities. J. Mol. Biol. 351: 66–75. 16002087 10.1016/j.jmb.2005.05.065 PMC3034352

[33] Huang CY, Chang YW, Chen WT (2008) Crystal structure of the N-terminal domain of *Geobacillus kaustophilus* HTA426 DnaD protein. Biochem. Biophys. Res. Commun. 375: 220–224. 10.1016/j.bbrc.2008.07.160 18703019

[34] Schneider S, Zhang W, Soultanas P, Paoli M (2008) Structure of the N-terminal oligomerization domain of DnaD reveals a unique tetramerization motif and provides insights into scaffold formation. J. Mol. Biol. 376: 1237–1250. 10.1016/j.jmb.2007.12.045 18206906 PMC3034642

[35] Marston FY, Grainger WH, Smits WK, Hopcroft NH, Green M, Hounslow AM, et al (2010) When simple sequence comparison fails: the cryptic case of the shared domains of the bacterial replication initiation proteins DnaB and DnaD. Nucleic Acids Res. 38: 6930–6942. 10.1093/nar/gkq465 20587500 PMC2978336

[36] Huang YH, Huang CC, Chen CC, Yang KJ, Huang CY (2015) Inhibition of *Staphylococcus aureus* PriA helicase by flavonol kaempferol. Protein J. 34: 169–172. 10.1007/s10930-015-9609-y 25894858 PMC7088215

[37] Wang CC, Tsau HW, Chen WT, Huang CY (2010) Identification and characterization of a putative dihydroorotase, KPN01074, from *Klebsiella pneumoniae*. Protein J. 29: 445–452. 10.1007/s10930-010-9272-2 20676924

[38] Huang CY, Hsu CH, Sun YJ, Wu HN, Hsiao CD (2006) Complexed crystal structure of replication restart primosome protein PriB reveals a novel single-stranded DNA-binding mode. Nucleic Acids Res. 34: 3878–3886. 16899446 10.1093/nar/gkl536 PMC1557812

[39] Huang YH, Lo YH, Huang W, Huang CY (2012) Crystal structure and DNA-binding mode of *Klebsiella pneumoniae* primosomal PriB protein. Genes Cells 17: 837–849. 10.1111/gtc.12001 22938024

[40] Huang YH, Lin MJ, Huang CY (2013) DnaT is a single-stranded DNA binding protein. Genes Cells 18: 1007–1019. 10.1111/gtc.12095 24118681

[41] Huang CY (2015) Inhibition of a putative dihydropyrimidinase from *Pseudomonas aeruginosa* PAO1 by flavonoids and substrates of cyclic amidohydrolases. PLoS One 10: e0127634 10.1371/journal.pone.0127634 25993634 PMC4437985

[42] Lo YH, Tsai KL, Sun YJ, Chen WT, Huang CY, Hsiao CD (2009) The crystal structure of a replicative hexameric helicase DnaC and its complex with single-stranded DNA. Nucleic Acids Res. 37: 804–814. 10.1093/nar/gkn999 19074952 PMC2647316

[43] Thompson AB, Calhoun AK, Smagghe BJ, Stevens MD, Wotkowicz MT, Hatziioannou VM, et al (2011) A gold nanoparticle platform for protein-protein interactions and drug discovery. ACS Appl. Mater. Interfaces 3: 2979–2987. 10.1021/am200459a 21699220

[44] Peng WF, Huang CY (2014) Allantoinase and dihydroorotase binding and inhibition by flavonols and the substrates of cyclic amidohydrolases. Biochimie 101: 113–122. 10.1016/j.biochi.2014.01.001 24418229

[45] Lohman TM, Tomko EJ, Wu CG (2008) Non-hexameric DNA helicases and translocases: mechanisms and regulation. Nat. Rev. Mol. Cell. Biol. 9: 391–401. 10.1038/nrm2394 18414490

[46] Bhattacharyya B, George NP, Thurmes TM, Zhou R, Jani N, Wessel SR, et al (2014) Structural mechanisms of PriA-mediated DNA replication restart. Proc. Natl. Acad. Sci. U. S. A. 111: 1373–1378. 10.1073/pnas.1318001111 24379377 PMC3910646

[47] Cadman CJ, Lopper M, Moon PB, Keck JL, McGlynn P (2005) PriB stimulates PriA helicase via an interaction with single-stranded DNA. J. Biol. Chem. 280: 39693–39700. 16188886 10.1074/jbc.M508521200

[48] Cadman CJ, McGlynn P (2004) PriA helicase and SSB interact physically and functionally. Nucleic Acids Res. 32: 6378–6387. 15576682 10.1093/nar/gkh980 PMC535688

[49] Koyama N, Inokoshi J, Tomoda H (2012) Anti-infectious agents against MRSA. Molecules 18: 204–224. 10.3390/molecules18010204 23262449 PMC6269750

[50] Otto M (2010) Basis of virulence in community-associated methicillin-resistant *Staphylococcus aureus*. Annu. Rev. Microbiol. 64: 143–162. 10.1146/annurev.micro.112408.134309 20825344

[51] Howden BP, Davies JK, Johnson PD, Stinear TP, Grayson ML (2010) Reduced vancomycin susceptibility in *Staphylococcus aureus*, including vancomycin-intermediate and heterogeneous vancomycin-intermediate strains: resistance mechanisms, laboratory detection, and clinical implications. Clin. Microbiol. Rev. 23: 99–139. 10.1128/CMR.00042-09 20065327 PMC2806658

[52] Bax BD, Chan PF, Eggleston DS, Fosberry A, Gentry DR, Gorrec F, et al (2010) Type IIA topoisomerase inhibition by a new class of antibacterial agents. Nature 466: 935–940. 10.1038/nature09197 20686482

[53] Kobayashi SD, DeLeo FR (2009) An update on community-associated MRSA virulence. Curr. Opin. Pharmacol. 9: 545–551. 10.1016/j.coph.2009.07.009 19726228

[54] Fischbach MA, Walsh CT (2009) Antibiotics for emerging pathogens. Science 325: 1089–1093. 10.1126/science.1176667 19713519 PMC2802854

[55] Chan KW, Lee YJ, Wang CH, Huang H, Sun YJ (2009) Single-stranded DNA-binding protein complex from *Helicobacter pylori* suggests an ssDNA-binding surface. J. Mol. Biol. 388: 508–519. 10.1016/j.jmb.2009.03.022 19285993

[56] Szymanski MR, Jezewska MJ, Bujalowski W (2010) Interactions of the *Escherichia coli* primosomal PriB protein with the single-stranded DNA. Stoichiometries, intrinsic affinities, cooperativities, and base specificities. J. Mol. Biol. 398: 8–25. 10.1016/j.jmb.2010.02.009 20156448 PMC3226724

[57] Drescher DG, Ramakrishnan NA, Drescher MJ (2009) Surface plasmon resonance (SPR) analysis of binding interactions of proteins in inner-ear sensory epithelia. Methods Mol. Biol. 493: 323–343. 10.1007/978-1-59745-523-7_20 18839357 PMC2864718

