# Supplementary figures and images for: Characterization of Staphylococcus aureus Primosomal DnaD Protein: Highly Conserved C-Terminal Region Is Crucial for ssDNA and PriA Helicase Binding but Not for DnaA Protein-Binding and Self-Tetramerization
Source: PLoS One. 2016 Jun 15;11(6):e0157593. doi: 10.1371/journal.pone.0157593 (PMC4909229; doi:10.1371/journal.pone.0157593)

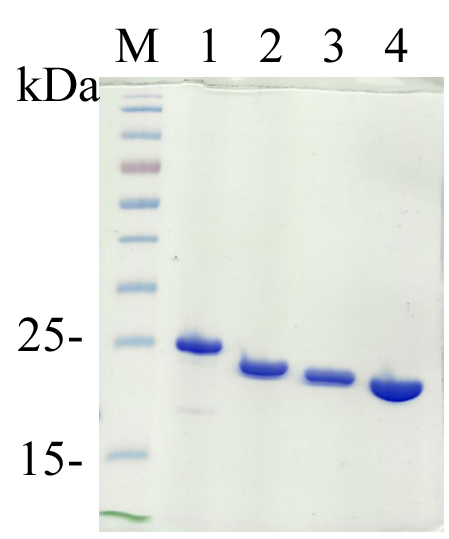

Supplement: S1 Fig — Coomassie Blue-stained SDS-PAGE (12%) of the purified SaDnaD (lane 1), SaDnaD1-204 (lane 2), SaDnaD1-200 (lane 3), SaDnaD1-195 (lane 4) and molecular mass standards (M) are shown. The sizes of the standard proteins, from the top down, are as follows: 170, 130, 100, 70, 55, 40, 35, 25, 15, and 10 kDa. (TIF) [file pone.0157593.s001.tif]

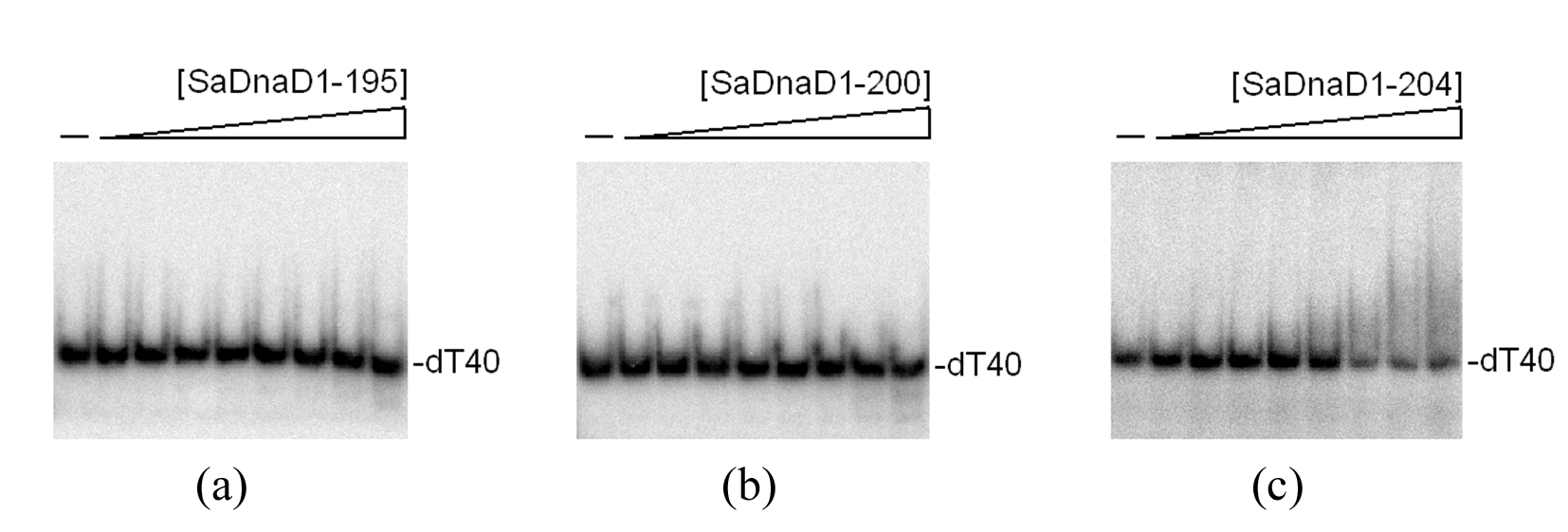

Supplement: S2 Fig — (A) SaDnaD1-195, (B) SaDnaD1-200, or (C) SaDnaD1-204, was incubated at 25°C for 30 min with 1.7 nM dT40 in a total volume of 10 μL in 20 mM Tris—HCl (pH 8.0) and 100 mM NaCl. The resulting samples were mixed with gel-loading solution (0.25% bromophenol blue and 40% sucrose; w/v), resolved on a native 8% polyacrylamide gel (8.3 × 7.3 cm) at 4°C in TBE buffer for 1–1.5 h at 100 V, and were visualized by phosphorimaging. The phosphor storage plate was scanned, and the data for complex and free DNA bands were digitized for quantitative analysis. (TIF) [file pone.0157593.s002.tif]

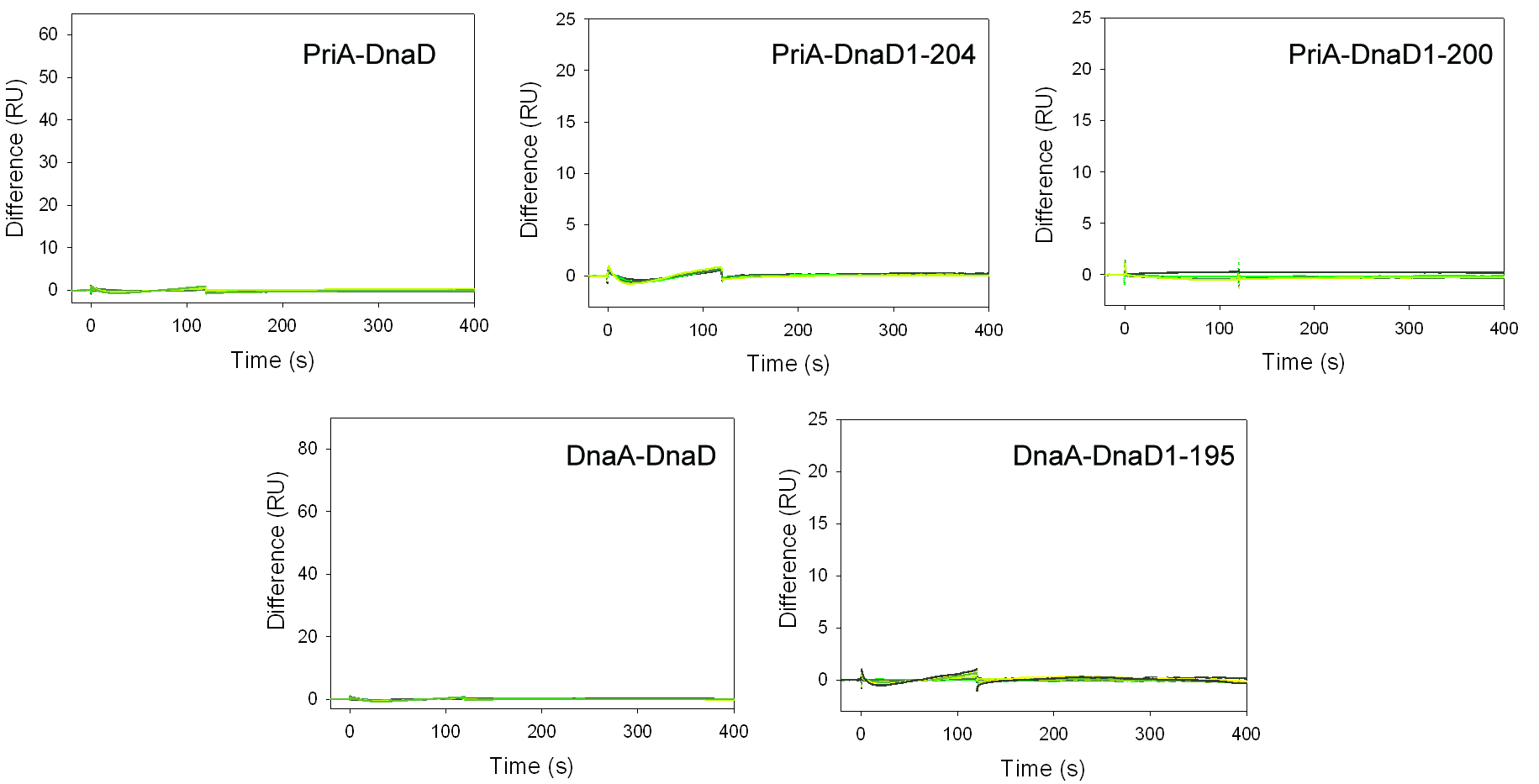

Supplement: S3 Fig — The estimated Kd values were derived by fitting the association and dissociation signals with a 1:1 (Langmuir) model using the Biacore T200 Evaluation Software. These residual plots were the calculated difference between the experimental and fitted data [57] for PriA—DnaD, PriA—DnaD1-204, PriA-DnaD1-200, DnaA—DnaD, and DnaA—DnaD1-195, respectively. (TIF) [file pone.0157593.s003.tif]

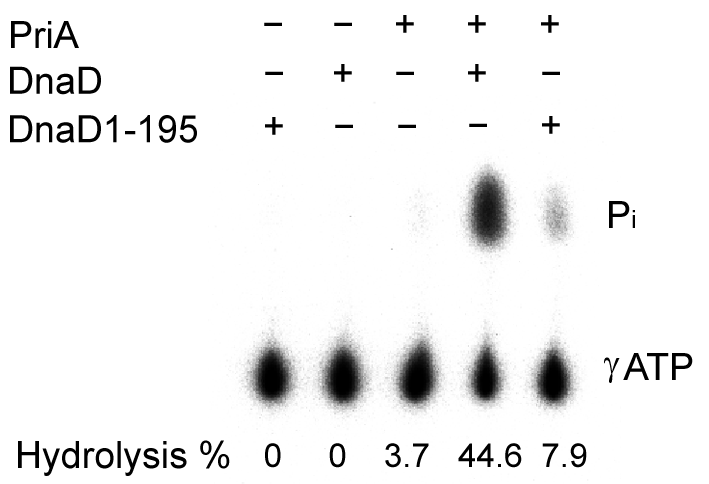

Supplement: S4 Fig — SaPriA ATPase assay was performed with 0.4 mM [γ-32P] ATP, 0.125 μM of SaPriA, and 0.1 μM PS4/PS3-dT25 DNA substrate for 1 h. To study the effect, SaDnaD (10 μM) or SaDnaD1-195 (10 μM) was added into the assay solution. Aliquots (5 μL) were taken and spotted onto a polyethyleneimine cellulose thin-layer chromatography plate, which was subsequently developed in 0.5 M formic acid and 0.25 M LiCl for 30 m. Reaction products were visualized by autoradiography and quantified with a Phosphorimager. (TIF) [file pone.0157593.s004.tif]

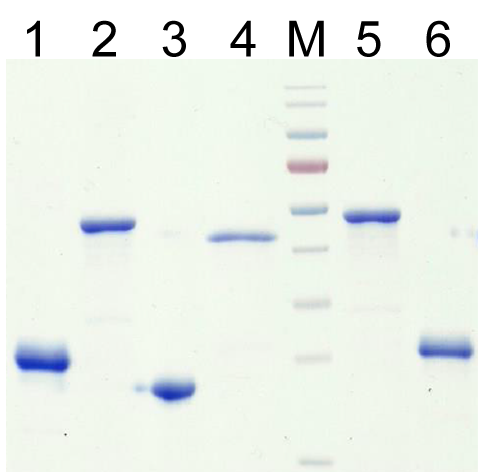

Supplement: S5 Fig — To obtain proteins without a His tag, tag-free SaDnaD, SaDnaDY176A, and SaDnaD1-195 proteins were produced using GST fusion and Factor Xa and were used in succeeding analysis. Coomassie Blue-stained SDS-PAGE (12%) of the purified SaDnaD (lane 1), GST-SaDnaD (lane 2), SaDnaD1-195 (lane 3), GST-SaDnaD1-195 (lane 4), GST-SaDnaDY176A (lane 5), SaDnaDY176A (lane 6), and molecular mass standards (M) are shown. The sizes of the standard proteins, from the top down, are as follows: 170, 130, 100, 70, 55, 40, 35, 25, and 15 kDa. (TIF) [file pone.0157593.s005.tif]

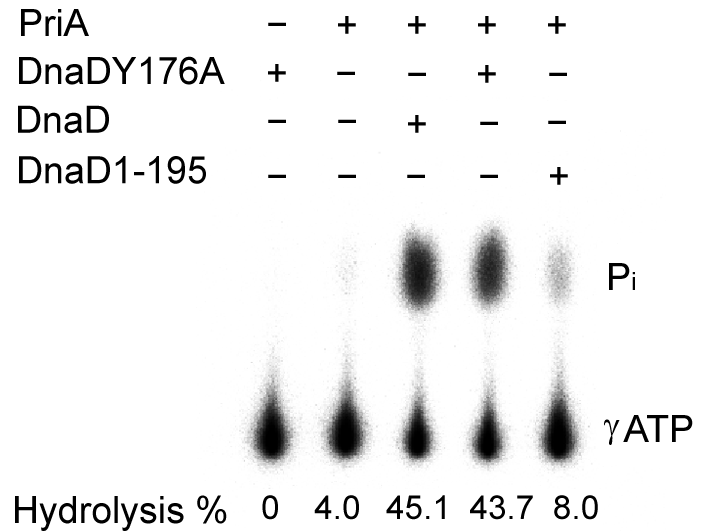

Supplement: S6 Fig — In this experiment, tag-free SaDnaD proteins were used for analysis of SaPriA ATPase activity. SaPriA ATPase assay was performed with 0.4 mM [γ-32P] ATP, 0.125 μM of SaPriA, and 0.1 μM PS4/PS3-dT25 DNA substrate for 1 h. To study the effect, SaDnaD (10 μM), SaDnaDY176A (10 μM), or SaDnaD1-195 (10 μM) was added into the assay solution. Aliquots (5 μL) were taken and spotted onto a polyethyleneimine cellulose thin-layer chromatography plate, which was subsequently developed in 0.5 M formic acid and 0.25 M LiCl for 30 m. Reaction products were visualized by autoradiography and quantified with a Phosphorimager. (TIF) [file pone.0157593.s006.tif]
